# The Immunometabolomic Interface Receptor Hydroxycarboxylic Acid Receptor 2 Mediates the Therapeutic Effects of Dimethyl Fumarate in Autoantibody-Induced Skin Inflammation

**DOI:** 10.3389/fimmu.2018.01890

**Published:** 2018-08-14

**Authors:** Melanie Wannick, Julian C. Assmann, Jakob F. Vielhauer, Stefan Offermanns, Detlef Zillikens, Christian D. Sadik, Markus Schwaninger

**Affiliations:** ^1^Institute for Experimental and Clinical Pharmacology and Toxicology, University of Lübeck, Lübeck, Germany; ^2^Department of Pharmacology, Max Planck Institute for Heart and Lung Research, Bad Nauheim, Germany; ^3^Department of Dermatology, Allergy, and Venereology, University of Lübeck, Lübeck, Germany

**Keywords:** pemphigoid disease, G protein-coupled receptor, immunomodulatory therapy, autoimmune blistering skin disease, neutrophils

## Abstract

The drug dimethyl fumarate (DMF) is in clinical use for the treatment of psoriasis and multiple sclerosis. In addition, it has recently been demonstrated to ameliorate skin pathology in mouse models of pemphigoid diseases, a group of autoimmune blistering diseases of the skin and mucous membranes. However, the mode of action of DMF in inflammatory skin diseases has remained elusive. Therefore, we have investigated here the mechanisms by which DMF improves skin pathology, using the antibody transfer model of bullous pemphigoid-like epidermolysis bullosa acquisita (EBA). Experimental EBA was induced by transfer of antibodies against collagen VII that triggered the infiltration of immune cells into the skin and led to inflammatory skin lesions. DMF treatment reduced the infiltration of neutrophils and monocytes into the skin explaining the improved disease outcome in DMF-treated animals. Upon ingestion, DMF is converted to monomethyl fumarate that activates the hydroxycarboxylic acid receptor 2 (HCA_2_). Interestingly, neutrophils and monocytes expressed *Hca2*. To investigate whether the therapeutic effect of DMF in EBA is mediated by HCA_2_, we administered oral DMF to *Hca2*-deficient mice (*Hca2*^−/−^) and wild-type littermates (*Hca2*^+/+^) and induced EBA. DMF treatment ameliorated skin lesions in *Hca2*^+/+^ but not in *Hca2*^−/−^ animals. These findings demonstrate that HCA_2_ is a molecular target of DMF treatment in EBA and suggest that HCA_2_ activation limits skin pathology by inhibiting the infiltration of neutrophils and monocytes into the skin.

## Introduction

Dimethyl fumarate (DMF) is an oral, immunomodulatory drug licensed for the treatment of multiple sclerosis (MS) and for moderate-to-severe plaque psoriasis. Upon oral ingestion, DMF is converted in the gut to monomethyl fumarate (MMF), which is the active principle of oral DMF treatment (1). Because of its overall favorable safety profile and its high efficacy, DMF has substantially improved the treatment of both MS and plaque psoriasis and has become a mainstay in the treatment of both diseases (2, 3). The mode of action of DMF in both plaque psoriasis and MS is only poorly understood.

Diverse biochemical actions of DMF have been uncovered, indicating that DMF may exert multiple immunomodulatory effects possibly contributing to its therapeutic effects. Among others, MMF was demonstrated to covalently modify cysteinyl residues of proteins by addition of a 2-monomethyl succinyl group, thereby activating the antioxidant nuclear factor erythroid 2-related factor 2 (NRF2) and inhibiting the glycolytic enzyme glyceraldehyde 3-phosphate dehydrogenase (GAPDH) (4, 5). In addition, MMF is an agonist of the G protein-coupled receptor hydroxycarboxylic acid receptor 2 (HCA_2_/GPR109A) (6). Recently, the agonism of MMF at HCA_2_ has been revealed to contribute to its therapeutic effects in murine experimental autoimmune encephalitis (EAE), a model for MS (7). In this study, oral DMF treatment reduced the number of infiltrating neutrophils in the spinal cord, and MMF impaired the migration and adhesion of neutrophils in a HCA_2_-dependent manner, indicating that the therapeutic effect of DMF in MS may be partially due to an inhibition of neutrophil recruitment into the CNS. The latter is a mechanism that has recently been suggested to be a key process in EAE and MS (8–11). HCA_2_ is expressed on neutrophils, monocytes, macrophages, and Langerhans cells (12). Its natural ligands are butyrate, hydroxy butyrate, and nicotinic acid. Thus, it belongs to the group of G protein-coupled receptors for short chain fatty acids, which have been uncovered to modify the course of disease of several autoimmune, autoinflammatory, and allergic diseases (13).

Pemphigoid diseases are a group of autoimmune blistering skin diseases caused by autoantibody formation against different proteins at the dermal–epidermal junction and the consequent recruitment of neutrophils into the skin (14). A recent study showed that DMF is beneficial in a preclinical model of bullous pemphigoid-like epidermolysis bullosa acquisita (EBA) (15), a variant of pemphigoid disease caused by autoantibodies directed to type VII collagen in the dermal–epidermal adhesion complex (14). This finding has led to a currently running clinical trial examining the efficacy of DMF in the most common pemphigoid disease bullous pemphigoid. However, the mode of action of DMF in pemphigoid diseases has remained elusive. Therefore, we have investigated here the contribution of HCA_2_ activation to the therapeutic effects of DMF in the antibody transfer mouse model of EBA (“experimental EBA”). Our study confirms the therapeutic effect of DMF in that model. Furthermore, we reveal that this therapeutic effect largely depends on the activation of HCA_2_, thus, highlighting HCA_2_ activation as new potential therapeutic principle in the treatment of pemphigoid diseases.

## Results

### DMF Reduces the Antibody-Induced Inflammatory Cell Infiltration in Experimental EBA

**Figure 1:**
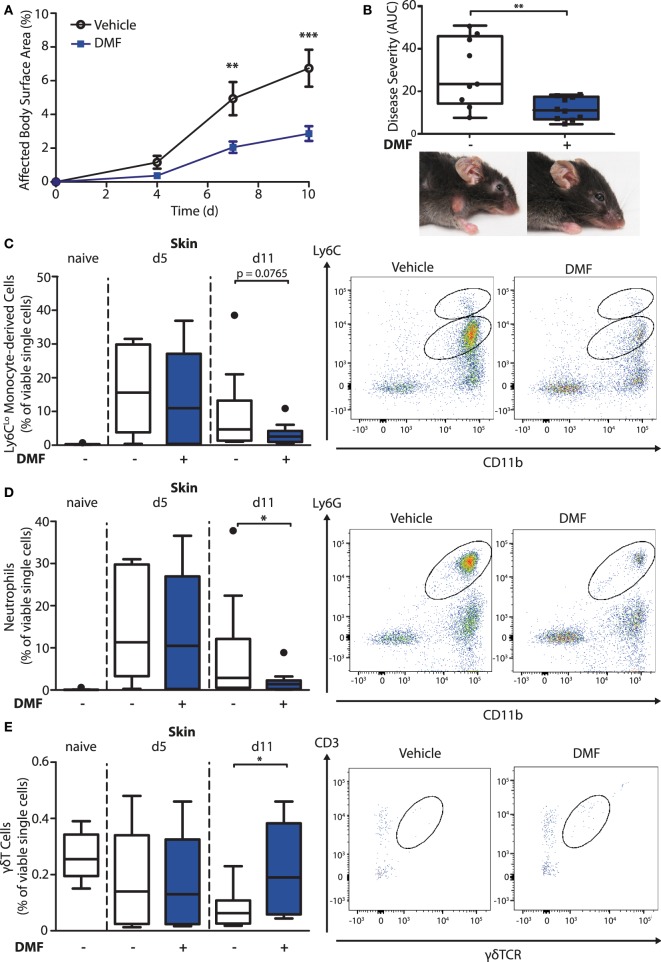
Dimethyl fumarate (DMF) treatment diminishes disease severity of experimental epidermolysis bullosa acquisita (EBA) by reducing elevated numbers of pro-inflammatory cells in the skin. **(A)** Clinical course of antibody transfer EBA in wild-type mice that were treated with DMF (50 mg/kg, p.o., twice per day) or vehicle. Two-way ANOVA, *F*(1/51) = 8.85, **p < 0.01; ***p < 0.001 (*n* = 9–14 mice, Bonferroni *post hoc* test). **(B)** Disease severity, calculated as area under the curve of the data in **(A)**, and clinical presentation. **p < 0.01 (Mann–Whitney test). **(C–E)** Quantification of immune cell populations in ear skin of naive mice or of animals on day 5 (d5) and 11 (d11) after first antibody injection. The numbers of cells are shown in percent of viable cells for **(C)** CD45^+^CD11b^+^Ly6C^Lo^ monocytes, **(D)** CD45^+^CD11b^+^Ly6G^+^ neutrophils, and **(E)** CD45^+^CD3^+^γδTCR^+^ γδT cells. Representative dot plots of day 11 are shown. *p < 0.05 (*n* = 9–14 mice, Mann–Whitney test).

To investigate the mode of action of DMF in inflammatory skin diseases, we induced EBA by transferring anti-collagen VII antibodies to mice. This mouse model reflects specifically the effector phase of the autoantibody-mediated skin disease. First, we set out to reproduce the protective effect of DMF that had been reported previously (15). We administered vehicle or DMF (50 mg/kg, twice daily, p.o.) to C57BL/6 mice starting 2 days prior to EBA induction. DMF significantly inhibited the precipitation of inflammatory skin lesions, thus, reducing disease severity at its peak by approximately 60% (Figures 1A,B).

**Figure 2:**
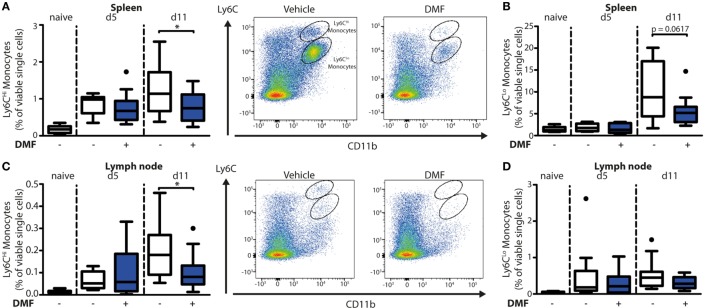
Dimethyl fumarate (DMF) treatment of mice inhibits trafficking of Ly6C^Hi^ monocytes to secondary lymphoid tissues. Quantification of CD45^+^CD11b^+^Ly6C^Hi^ and CD45^+^CD11b^+^Ly6C^Lo^ monocyte populations in spleen **(A,B)** and lymph nodes **(C,D)** of naive mice or of animals on day 5 (d5) and 11 (d11) after first antibody injection. The animals received either vehicle or DMF (50 mg/kg, p.o., twice per day). The numbers of cells are shown in percent of viable single cells. Representative dot plots of day 11 are shown. *p < 0.05 (*n* = 9–14 mice, Mann–Whitney test).

To uncover the mode of action of DMF in EBA, we next characterized its effects on immune cell numbers in the lesional skin, peripheral blood, and lymphoid tissues by flow cytometry. In this profiling, we distinguished neutrophils as CD45^+^CD11b^+^Ly6G^+^ cells and monocyte-derived cells as CD45^+^CD11b^+^Ly6C^+^ cells. The latter are a heterogeneous population in skin comprised of monocyte-derived Langerhans cells, dendritic cells, and macrophages (16, 17). At disease onset (day 5 after EBA induction), the relative numbers of neutrophils and CD11b^+^Ly6C^Lo^ monocyte-derived cells were similar in the skin of vehicle- and DMF-treated mice (Figures 1C,D). However, at a more advanced disease stage (day 11 after EBA induction), DMF treatment diminished the relative number of neutrophils. In addition, the lesional skin of DMF-treated animals showed a trend toward lower relative numbers of CD11b^+^Ly6C^Lo^ monocyte-derived cells at day 11 after the first antibody transfer (Figures 1C,D). In blood, neither neutrophils nor CD11b^+^Ly6C^Lo^ monocytes were affected by DMF treatment (Figures S1 and S2 in Supplementary Material). While DMF treatment had no significant effect on relative numbers of CD11b^+^Ly6C^Hi^ monocytes in the peripheral blood and of CD11b^+^Ly6C^Hi^ monocyte-derived cells in the skin, it reduced CD11b^+^Ly6C^Hi^ monocytes in the spleen and in lymph nodes by day 11 after the induction of EBA (Figures 2A,C; Figure S2 in Supplementary Material). The relative numbers of CD11b^+^Ly6C^Lo^ monocytes and neutrophils were not affected by DMF treatment in lymphoid tissue (Figures 2B,D; Figure S1 in Supplementary Material).

In contrast to the partial reduction of myeloid cells, the relative number of CD3-NK1.1^+^ natural killer cells that could not be detected in skin was unchanged upon EBA induction and not affected by DMF treatment in blood and lymphoid tissue (Figure S3 in Supplementary Material). Regarding the lymphoid cells, the relative numbers of CD45^+^γδTCR^+^ T cells in the skin was higher in DMF-treated than in vehicle-treated animals on day 11 after EBA induction (Figure 1E). In secondary lymphoid tissues and in blood, DMF treatment had no effect on γδT cells (Figure S4 in Supplementary Material). Interestingly, the relative number of CD3^+^ γδTCR^−^ αβT cells was increased in DMF-treated mice in spleen, but not in lymph nodes, blood, and skin (Figure S5 in Supplementary Material). The increased numbers of αβT cells in spleen could represent regulatory T cells that were shown to dampen disease progression in EBA (18). Overall, the data indicate that DMF modulates the numbers of neutrophils, CD11b^+^Ly6C^Lo^ monocyte-derived cells, and γδT cells in the skin.

### DMF Treatment Increases CD62L Levels on Neutrophils and CD11b^+^Ly6C^Lo^ Monocytes

**Figure 3:**
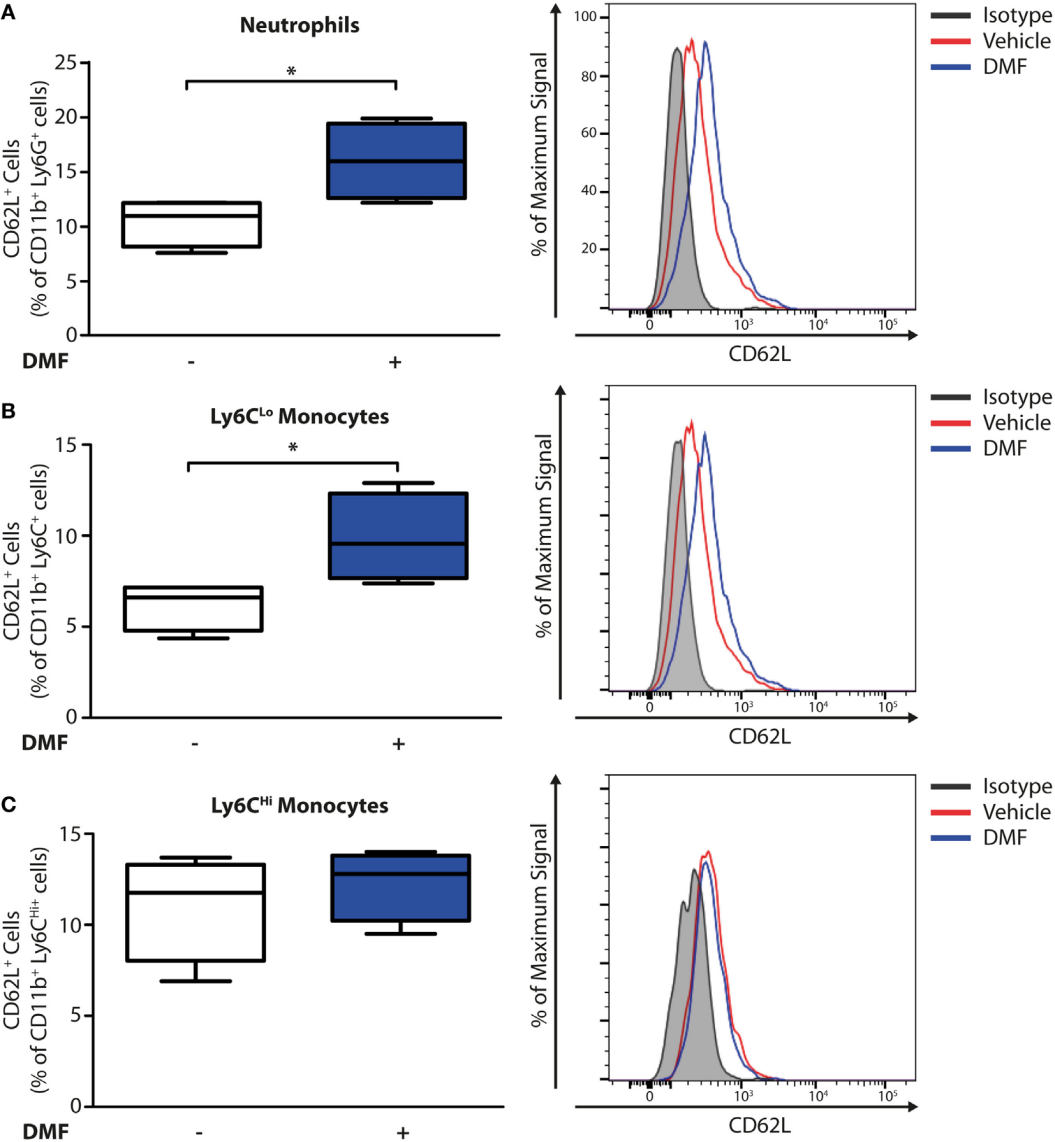
Dimethyl fumarate (DMF) treatment of mice inhibits activation of blood neutrophils and Ly6C^Lo^ monocytes. Quantification of CD62L on immune cell populations in blood of wild-type mice on day 11 after first antibody injection. CD62L levels are decreased during activation and tissue infiltration. The numbers of CD62L^+^ cells are shown in percent of **(A)** CD45^+^CD11b^+^Ly6G^+^ neutrophils, **(B)** CD45^+^CD11b^+^Ly6C^Lo^ monocytes, and **(C)** CD45^+^CD11b^+^Ly6C^Hi^ monocytes. Representative histograms show isotype controls (gray) as well as anti-CD62L stained samples of vehicle- (red) and DMF-treated mice (blue). *p < 0.05 (*n* = 4 mice, Mann–Whitney test).

Having established that DMF treatment decreases the numbers of neutrophils and CD11b^+^Ly6C^Lo^ monocyte-derived cells in skin lesions, we addressed whether these effects may be due to an inhibition of recruitment into the skin. Non-activated neutrophils and monocytes express high levels of CD62L (L-selectin) on their surface that is cleaved during activation and trans-endothelial migration. Consequently, low levels of CD62L are a marker of activated or migrating cells (19). Therefore, we determined the CD62L levels on immune cells in the blood on day 11 of the EBA model by flow cytometry. Neutrophils and CD11b^+^Ly6C^Lo^ monocytes but not CD11b^+^Ly6C^Hi^ monocytes (Figure 3) expressed higher levels of CD62L, thus being not activated or transmigrating, upon DMF treatment. This indicates that DMF lowers the infiltration of neutrophils and CD11b^+^Ly6C^Lo^ monocytes and ameliorates EBA pathology by impairing the trans-endothelial migration. Furthermore, CD62L is essential for migration through high endothelial venules of secondary lymphoid organs (20). Indeed, upon DMF treatment, neutrophils and monocytes that entered lymphoid tissue had similar level of CD62L (Figure S6 in Supplementary Material).

### Experimental EBA Increases HCA_2_ Expression in the Blood and the Skin

**Figure 4:**
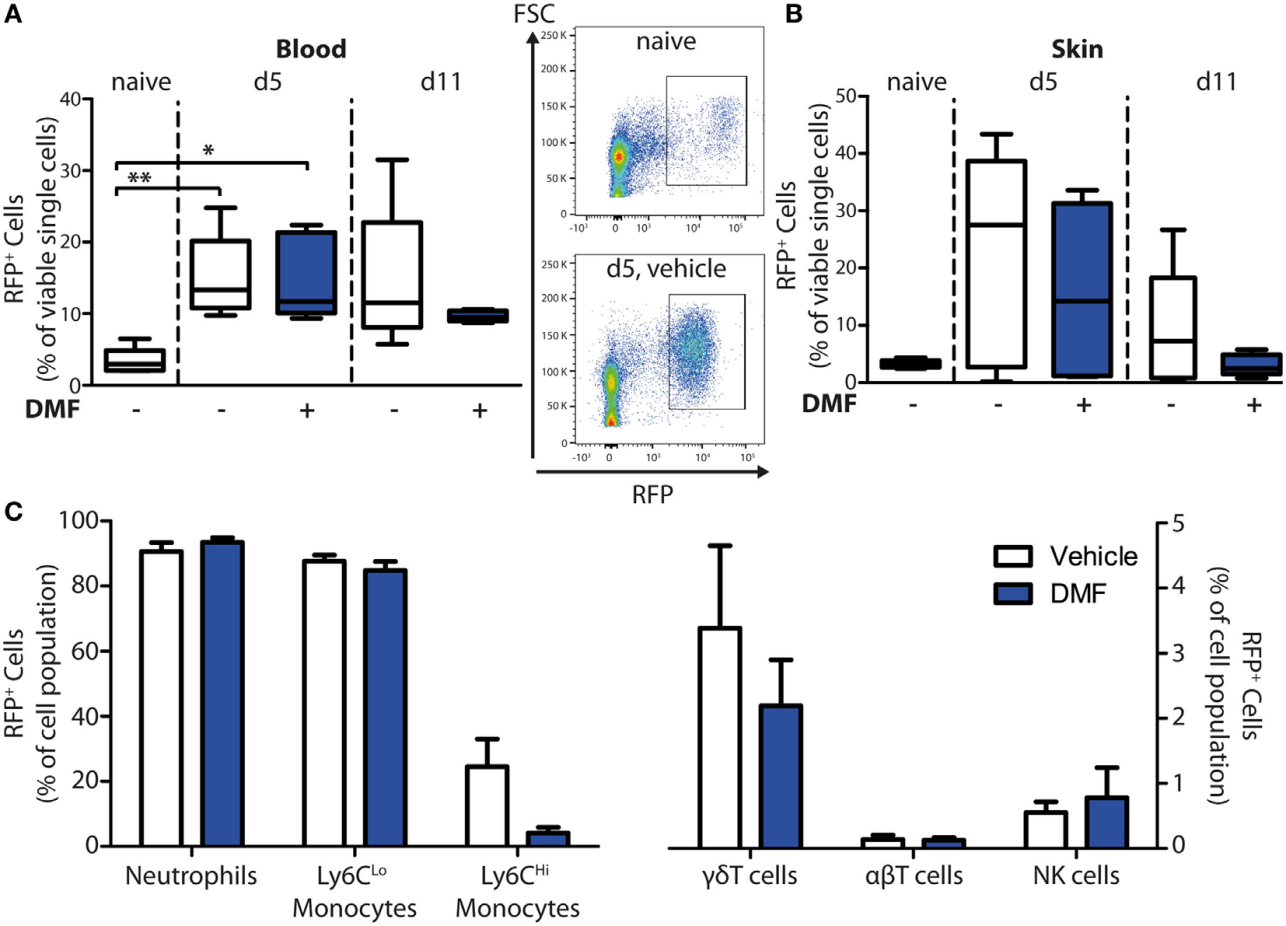
HCA_2_ expression increases in blood and skin upon induction of experimental epidermolysis bullosa acquisita. Quantification of monomeric red fluorescent protein (mRFP)^+^ cells in blood and ear skin of *Hca2*^mRFP^ mice. The numbers of RFP^+^ cells are shown in percent of viable cells for **(A)** blood and **(B)** skin of naive mice or of animals on day 5 (d5) and 11 (d11) after first antibody injection. Mice were treated with vehicle of dimethyl fumarate (DMF) (50 mg/kg, p.o., twice per day). *p < 0.05; **p < 0.01 (*n* = 5 mice, Kruskal–Wallis Test with Dunn’s *post hoc* test). **(A)** Representative dot plots from naïve mice and animals at d5 (vehicle-treated group) are shown. **(C)** Quantification of mRFP^+^ cells in immune cell populations of DMF- and vehicle-treated mice at d11. The numbers of mRFP^+^ cells are expressed as percent of CD45^+^CD11b^+^Ly6G^+^ neutrophils, CD45^+^CD11b^+^Ly6C^Lo^ monocytes, CD45^+^CD11b^+^Ly6C^Hi^ monocytes, CD45^+^CD3^+^γδTCR^+^ γδT cells, CD45^+^CD3^+^γδTCR^−^ αβT cells, and CD45^+^CD3^−^NK1.1^+^ NK cells. Means ± SEM are depicted (*n* = 5 mice).

To determine whether DMF may exert its therapeutic effects in EBA through activation of HCA_2_ on infiltrating cell populations, we profiled the spatiotemporal dynamics of *Hca2* expression in the peripheral blood and in the skin in the course of experimental EBA. For this purpose, we employed the *Hca2*^mRFP^ (*Gpr109a*^mRFP^) reporter mouse line, in which the *Hca2* locus directs the expression of the monomeric red fluorescent protein (mRFP) (21), and assayed mRFP expression by FACS. This approach revealed that significantly more immune cells in blood were mRFP^+^ after EBA induction than in naïve mice (Figure 4A). In parallel, the relative numbers of mRFP^+^ cells increased in the skin upon induction of EBA (Figure 4B). Nearly all neutrophils and CD11b^+^Ly6C^Lo^ monocytes expressed the receptor, whereas only 5–20% of CD11b^+^Ly6C^Hi^ monocytes were mRFP^+^ (Figure 4C). Among T cells, we detected a small subpopulation of γδT cells that were mRFP^+^, thus, providing a possible explanation for their responsiveness to DMF treatment. The increase of mRFP^+^ cells in the blood and the skin in response to EBA induction is probably due to a rise of mRFP^+^ neutrophils and CD11b^+^Ly6C^Lo^ monocytes in blood (Figure S1 in Supplementary Material) and their infiltration into skin lesions (22). The percentage of mRFP^+^ cells among immune cells in the blood and the skin remained stable even under DMF treatment (Figures 4A,B).

### The Therapeutic Effect of DMF in EBA Is HCA_2_-Dependent

**Figure 5:**
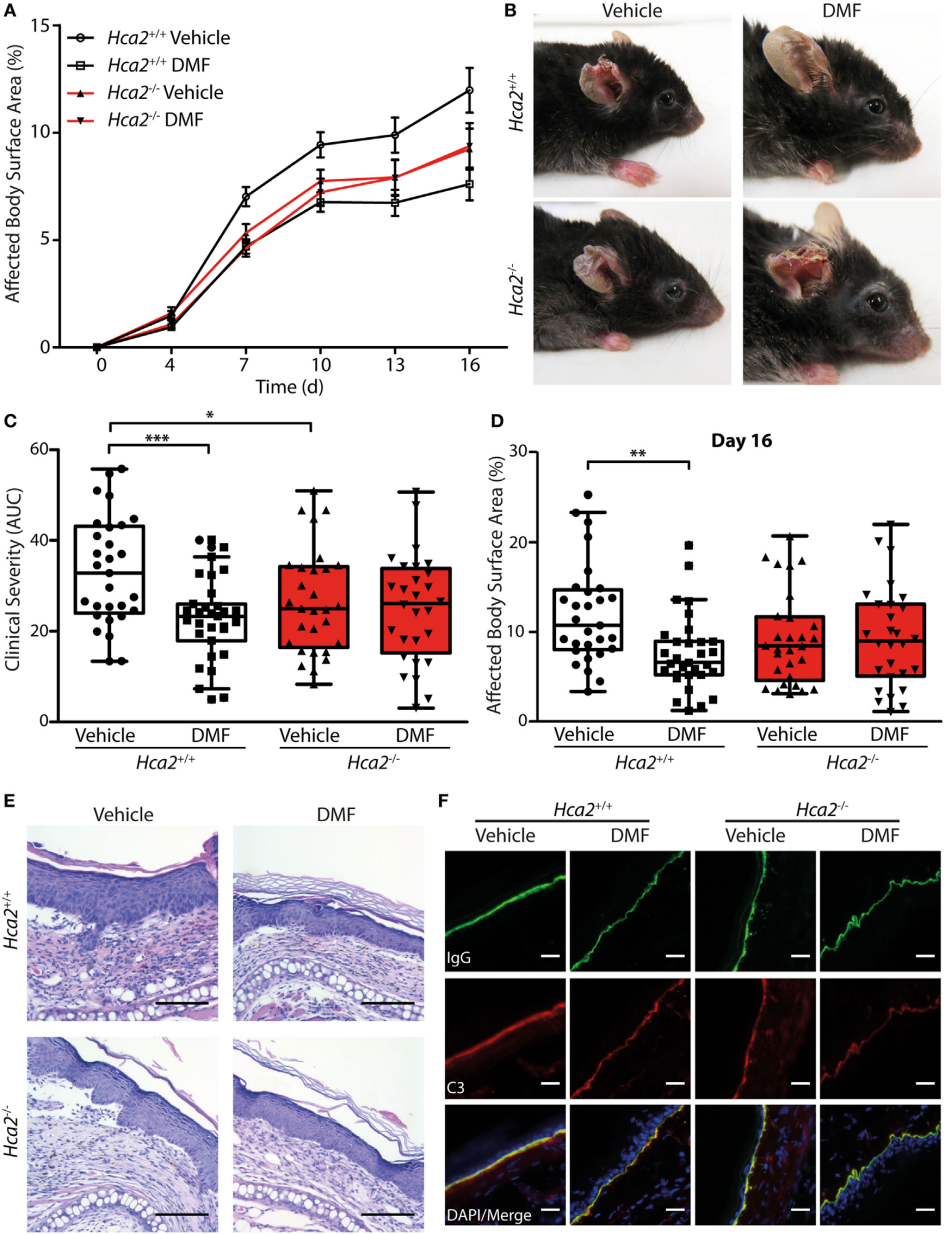
Therapeutic dimethyl fumarate (DMF) effects in experimental epidermolysis bullosa acquisita (EBA) depend on HCA_2_. **(A)** Clinical course of antibody transfer EBA in *Hca2*^+/+^ and *Hca2*^−/−^ mice that received oral vehicle or DMF treatment (50 mg/kg, p.o., twice per day). **(B)** Representative clinical presentation in the four experimental groups. **(C)** Clinical severity, calculated as area under the curve of the data in **(A)**. Two-way ANOVA, interaction between genotype and treatment *F*(1/113) = 4.94, *p < 0.05; ***p < 0.001 (*n* = 28–31 mice, Bonferroni *post hoc* test) **(D)** Affected body surface area on day 16. ANOVA, interaction between genotype and treatment *F*(1/113) = 5.75, **p < 0.01 (*n* = 28–31 mice, Bonferroni *post hoc* test). **(E)** Representative pictures of hematoxylin- and eosin-stained skin sections. Scale bar = 100 µm. **(F)** Representative immunohistochemical staining of anti-collagen VII-IgG and C3 depositions at the dermal–epidermal junction. Scale bar = 20 µm.

After oral ingestion, DMF is converted to MMF that activates HCA_2_ (6). The finding that HCA_2_ is expressed by neutrophils and CD11b^+^Ly6C^Lo^ monocyte-derived cells that respond to oral DMF treatment (Figure 1) is compatible with the idea that the receptor is required for the therapeutic efficacy of DMF. To directly test this concept, we investigated whether the therapeutic effect of DMF in EBA depends on HCA_2_. For this purpose, we induced EBA in *Hca2*^−/−^ mice and analyzed the course of disease in comparison to *Hca2*^+/+^ littermates. While DMF treatment again reduced skin lesions in *Hca2*^+/+^ mice throughout the entire period of observation (area under the curve, AUC) and on individual days, it lacked a therapeutic effect in *Hca2*^−/−^ littermates (Figures 5A–D). Statistical analysis revealed that the effect of DMF depended on HCA_2_ expression.

Interestingly, we also found that the severity of skin inflammation was significantly reduced in vehicle control-treated *Hca2*^−/−^ mice compared to their vehicle-treated *Hca2*^+/+^ littermates when compared as AUC of diseases activity (Figure 5C), suggesting that long-term HCA_2_ deficiency could have an additional effect. However, at the end of the experiment (day 16 after EBA induction) the clinical score was similar between the genotypes in vehicle-treated groups (Figure 5D).

In fully developed lesions, histopathological inspection showed thickening of the inflamed epidermis and split formation in all groups (Figure 5E). Moreover, depositions of the injected anti-collagen VII-IgG and activated C3 factor of the complement cascade could be detected at the dermal–epidermal junction in all experimental groups providing evidence for the successful EBA induction (Figure 5F; Figure S7 in Supplementary Material).

In addition to activating HCA_2_, DMF and its metabolite MMF stimulate the anti-oxidative transcription factor NRF2 (4). In lesional skin of *Hca2*^+/+^ and *Hca2*^−/−^ mice, DMF treatment increased the expression of *Nqo*1, a known target gene of NRF2 (Figure S8 in Supplementary Material), excluding the possibility that the lack of efficacy of DMF in *Hca2*^−/−^ mice is due to differences in the tissue distribution of the active agent.

## Discussion

In this study, we have addressed the mode of action of DMF in pemphigoid diseases, a group of prototypical organ-specific, autoantibody- and neutrophil-driven disorders (14). Using the antibody transfer EBA mouse model, a bullous pemphigoid-like disease, we first confirmed the therapeutic effect of DMF in experimental pemphigoid diseases and then profiled the therapeutic effect of DMF in these diseases on the cellular and molecular level. On the cellular level, DMF treatment curbed the infiltration of the skin with neutrophils and monocytes. On the molecular level, we show that HCA_2_ is required for the therapeutic effect of DMF in experimental EBA. Upon DMF ingestion, HCA_2_ is activated by MMF, the active metabolite of DMF (6, 21). Our data corroborate previous reports that neutrophils and CD11b^+^Ly6c^Lo^ monocyte-derived cells express *Hca2* (23, 24). Interestingly, the numbers of these two cell populations in EBA skin lesions were reduced by DMF treatment. Apparently, this is due to a lower infiltration into the diseased skin because DMF treatment reduced the cleavage of CD62L that occurs during tissue infiltration of blood neutrophils and CD11b^+^Ly6C^Lo^ monocytes. Indeed, by activating HCA_2_ MMF is able to inhibit the adhesion and migration of neutrophils (7).

Could the inhibition of neutrophil and monocyte infiltration into the diseased skin be the mode of action by which DMF ameliorates EBA manifestations? In EBA and pemphigoid disease autoantibodies bind to the dermal–epidermal junction and trigger a complement activation that leads to the infiltration of neutrophils and monocytes (14, 25). Specifically, the complement factor C5a stimulates release of leukotriene B4 that seems to be a key chemoattractant of neutrophils in EBA (22). Ablating neutrophils or reducing leukotriene B4 synthesis ameliorated skin lesions in EBA. After infiltrating the skin, neutrophils release reactive oxygen species and seem to degrade the adhesion between dermis and epidermis (15, 26). Evidence for a functional role of monocyte-derived cells in EBA is still more circumstantial. Comparing the effect of two antibodies that deplete monocytes and neutrophils (anti-Ly6C/G) or only neutrophils (anti-Ly6G) suggests that the depletion of monocytes has an additional beneficial effect on experimental EBA (22, 26). Moreover, monocytes are able to execute subepidermal cleft formation in an *in vitro* model of the disease (25, 27). The roles of neutrophils and monocytes in pemphigoid diseases suggest that therapeutic principles targeting, like DMF, the recruitment of both neutrophils and monocytes into the skin may be superior to strategies solely targeting neutrophils.

In our study, the number of γδT cells in skin was significantly higher in DMF-treated animals. γδT cells have previously been described to promote disease development. Ablating these cells with an anti-γδTCR antibody led to a reduced disease severity in EBA (28). In contrast, mice treated with DMF showed a milder clinical course at the time when γδT cells were elevated in the skin. Importantly, γδT cells are a heterogeneous cell population. Although some γδT cells produce pro-inflammatory cytokines, such as IL-17 and IFN-γ, other subpopulations, including IL-10 expressing γδT cells, have been described as regulatory cells required for the differentiation of Treg cells (29, 30). HCA_2_^+^ γδT cells have not been reported previously. Thus, a future study thoroughly characterizing this subpopulation and the effect of HCA_2_ activation in these cells is warranted.

A key finding of our study is that in EBA the therapeutic DMF effect depends on HCA_2_ as has previously been reported in a mouse model of MS (7). In addition to neutrophils, monocyte-derived cells, γδT cells, and some other cell types in the skin express HCA_2_ and have been shown to be affected by HCA_2_ activation. Unexpectedly, vehicle-treated *Hca2*-deficient mice showed reduced disease activity when the AUC was analyzed (Figure 5C). A basal phenotype of *Hca2*-deficient mice has not been described so far in other models of (skin) autoimmune diseases. Considering the fact that endogenous compounds, such as butyrate, nicotinic acid, and β-hydroxybutyrate, function as agonists of HCA_2_, a basal activation of HCA_2_ in some body compartments is possible, even in the absence of DMF treatment. Why such a basal HCA_2_ activation should have the opposite effect of activation by DMF is unclear so far.

Apart from HCA_2_ activation, DMF has been shown to act on the anti-oxidative NRF2 signaling pathway and on the glycolytic enzyme GAPDH (4, 5). By stimulating NRF2, DMF treatment induces a range of anti-oxidative and anti-inflammatory genes, an effect that is independent of HCA_2_ (7). Whether HCA_2_-independent effects contribute to the therapeutic effects of DMF in pemphigoid disease is unclear so far. In any case, identification of HCA_2_ as an essential molecular target for EBA treatment suggests a strategy how to expand the therapeutic armamentarium for the treatment of autoimmune skin diseases. As a druggable G protein-coupled receptor, HCA_2_ is activated by numerous compounds that await testing in pemphigoid disease and other autoimmune disorders (31).

## Materials and Methods

### Mice

*Hca2*^−/−^ mice were generated on the *C57BL/6* background, which is highly susceptible to the induction of passive EBA (32, 33). *Hca2*^−/−^ and their respective littermate controls (*Hca2*^+/+^) used for experiments were 8- to 12-week-old and all experimental groups were age- and sex-matched. To control for cage-specific effects, *Hca2*^−/−^ and *Hca2*^+/+^ mice and both treatment groups were housed together in individually ventilated cages on a 12–12 h light cycle with *ad libitum* access to food and water. All animal experiments were performed in accordance with Animal Protection Law and were approved by the Animal Research Ethics Board of the Ministry of the Environment, Kiel, Germany [Ethics approval V 242-79898-2015 (110-8-15)].

### Generation of Anti-Collagen VII

To generate antibodies directed to murine collagen VII (“anti-collagen VII”), New Zealand White rabbits were immunized with 200 µg of a protein mixture containing three different recombinant proteins (“Col7A, B, and C”) derived from the non-collagenous 1 domain of type VII collagen together with incomplete Freund’s adjuvant, as described previously (33). IgGs were isolated from the serum of immunized rabbits by use of protein G, and afterward IgGs affinity purified with his-COL7 to specifically obtain rabbit anti-collagen VII IgG. To control for batch effects, all experiments were conducted using anti-COL7 IgG from at least two different batches.

### Induction of Autoantibody Transfer (“Passive”) EBA

Passive EBA was induced by i.p. injections of 75–100 µg affinity-purified anti-collagen VII IgG on day 0, 2, and 4 of the experiments. Disease severity was scored in a blinded fashion every 3 days for 16 days starting on day 4. The percentage of “affected skin” of the total body surface area of mice was assessed as described previously (18).

### DMF Treatment

Dimethyl fumarate (Sigma-Aldrich) was prepared daily and suspended in 0.8% Methocel™/H_2_O. Mice were treated with vehicle or DMF (50 mg/kg body weight) every 12 h by gavage.

### Flow Cytometry

For the flow cytometric analysis of cells from blood, skin, spleen, and inguinal lymph nodes during EBA, mice were deeply anesthetized with ketamine (0.1 mg/g)/xylazin (0.015 mg/g) and killed. Blood was drawn from the right ventricle of the heart before spleen, inguinal lymph nodes, and ear skin were collected and stored on ice for further processing.

Cells were isolated from blood using the erythrocyte lysing buffer (Qiagen) in compliance with the manufacturer’s instructions. Spleens and lymph nodes were homogenized using a 70-µm cell strainer (BD). Then, spleen cells underwent erythrocyte lysing as described above. Small pieces of ear skin were digested with liberase™ TL (1.2 mg/ml, Sigma) diluted in Iscove’s Modified Dulbecco’s Medium (Gibco) for 90 min at 37°C with constant agitation and subsequently ground on a 70-µm cell strainer. Single cells were resuspended in FACS buffer.

Using 2 × 10^6^ cells per staining, blocking was performed with an anti-CD16/32 antibody (1:100, Mouse BD Fc Block). Surface antigens were stained with the appropriate antibodies (see Table 1) and a viability dye (eBioscience™ Fixable Viability Dye eFluor™ 780 or 660). Stained cells were analyzed using a FACS LSRII system and FACS DIVA software (BD Biosciences). Analysis was performed using FlowJo 10.3 software. For the gating strategy of viable and CD45 expressing cells, see Figure S9 in Supplementary Material.

**Table 1:** Antibodies used for the flow cytometric analysis of immune cells.

Antibody	Clone	Supplier
Brilliant Violet 510™ anti-mouse CD45	30-F11	Biolegend
Brilliant Violet 650™ anti-mouse/human CD11b	M1/70	Biolegend
PE/Cy7 anti-mouse Ly-6C	HK1.4	Biolegend
PerCP/Cy5.5 anti-mouse Ly-6G	1A8	Biolegend
Brilliant Violet 421™ anti-mouse NK-1.1	PK136	Biolegend
FITC anti-mouse CD3	145-2C11	Biolegend
PerCP/Cy5.5 anti-mouse γδTCR	REA633	Miltenyi

### Immunohistochemistry

For the detection of skin-bound complement factor C3 and anti-collagen VII antibodies, cryosections (10 µm) were fixed with acetone for 20 min at −20°C, washed and subsequently blocked with 1% BSA in PBS for 1 h. The sections were incubated with an anti-C3 antibody (1:400, clone 11H9, Hycult Biotech) overnight at 4°C. After 3 washing steps with PBS, the appropriate secondary antibodies (Alexa 488-labeled anti-rat IgG, 1:400, ThermoFisher; Cy3-labeled anti-rabbit IgG, 1:400, Jackson ImmunoResearch) and DAPI (2 µg/ml) were added and incubated for 1 h at room temperature. Then, the sections were embedded in Mowiol-DABCO. Images were acquired using a Leica DMI6000B fluorescence microscope.

### Histology

Paraffin-embedded tissue sections (5 µm) were prepared on a microtome (Leica), dried overnight at room temperature and de-paraffinized using xylene and a descending alcohol dilution. The sections were incubated in 1% (v/v) acetic acid for 20 s before staining with hematoxylin (modified after Gill, Merck) for 10 min. Counter-staining with eosin Y (Merck) was carried out after two consecutive washes with warm tap water for 2 min. Then, the sections were dehydrated using an ascending alcohol series and two incubations with xylene before embedding in Eukitt embedding medium (Merck).

### Quantitative Real-Time PCR

RNA of frozen lesional skin was isolated using the Navy Bullet Lysis Kit (Next Advance) in compliance with the manufacturer’s instructions. cDNA synthesis was performed as previously described (7). The following primer sets were used: Ppib sense 5′-GGC TCC GTC GTC TTC CTT TT-3′, antisense 5′-ACT CGT CCT ACA GAT TCA TCT CC-3′, Nqo1 sense 5′-ATT CTC TGG CCG ATT CAG AGT G-3′, and antisense 5′-AGA CGG TTT CCA GAC GTT TCT T-3′.

### Statistical Analysis

All data showing time courses of disease development are represented as the mean ± SEM. Bar graphs are shown as Box-Whisker plots according to Tukey. The statistical analysis was carried out using Prism (version 5.0, GraphPad Software, San Diego, USA) *p < 0.05, **p < 0.01, and ***p < 0.001.

## Ethics Statement

All animal experiments of this study were in accordance with the recommendations of the German Animal Protection Law and approved by the local animal ethics committee (Ministerium für Landwirtschaft, Umwelt und ländliche Räume, Kiel, Germany).

## Author Contributions

MW, JA, DZ, CS, and MS contributed to the conception and design of the study. MW analyzed cells by flow cytometry. MW and JA performed mouse experiments. JV contributed to sample preparation and immunohistochemical stainings. SO provided valuable tools and conceptual background. MW, JA, and MS wrote the first draft of the manuscript. All authors contributed to manuscript revision, read and approved the submitted version.

## Conflict of Interest Statement

The authors declare that the research was conducted in the absence of any commercial or financial relationships that could be construed as a potential conflict of interest.
