# Supplementary figures and images for: The Immunometabolomic Interface Receptor Hydroxycarboxylic Acid Receptor 2 Mediates the Therapeutic Effects of Dimethyl Fumarate in Autoantibody-Induced Skin Inflammation
Source: Front Immunol. 2018 Aug 14;9:1890. doi: 10.3389/fimmu.2018.01890 (PMC6102353; doi:10.3389/fimmu.2018.01890)

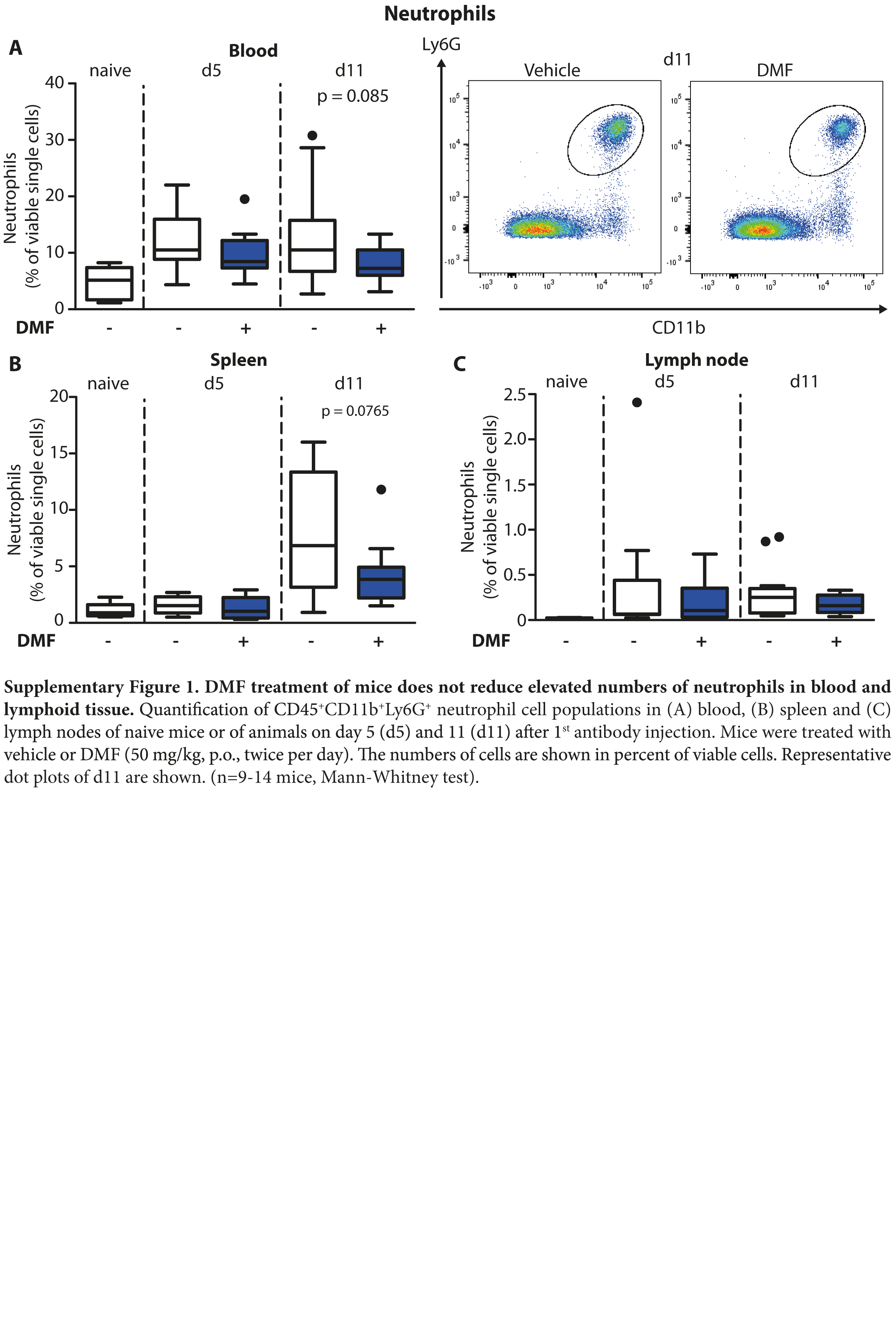

Supplement: Supplementary file 1 [file image_1.tif]

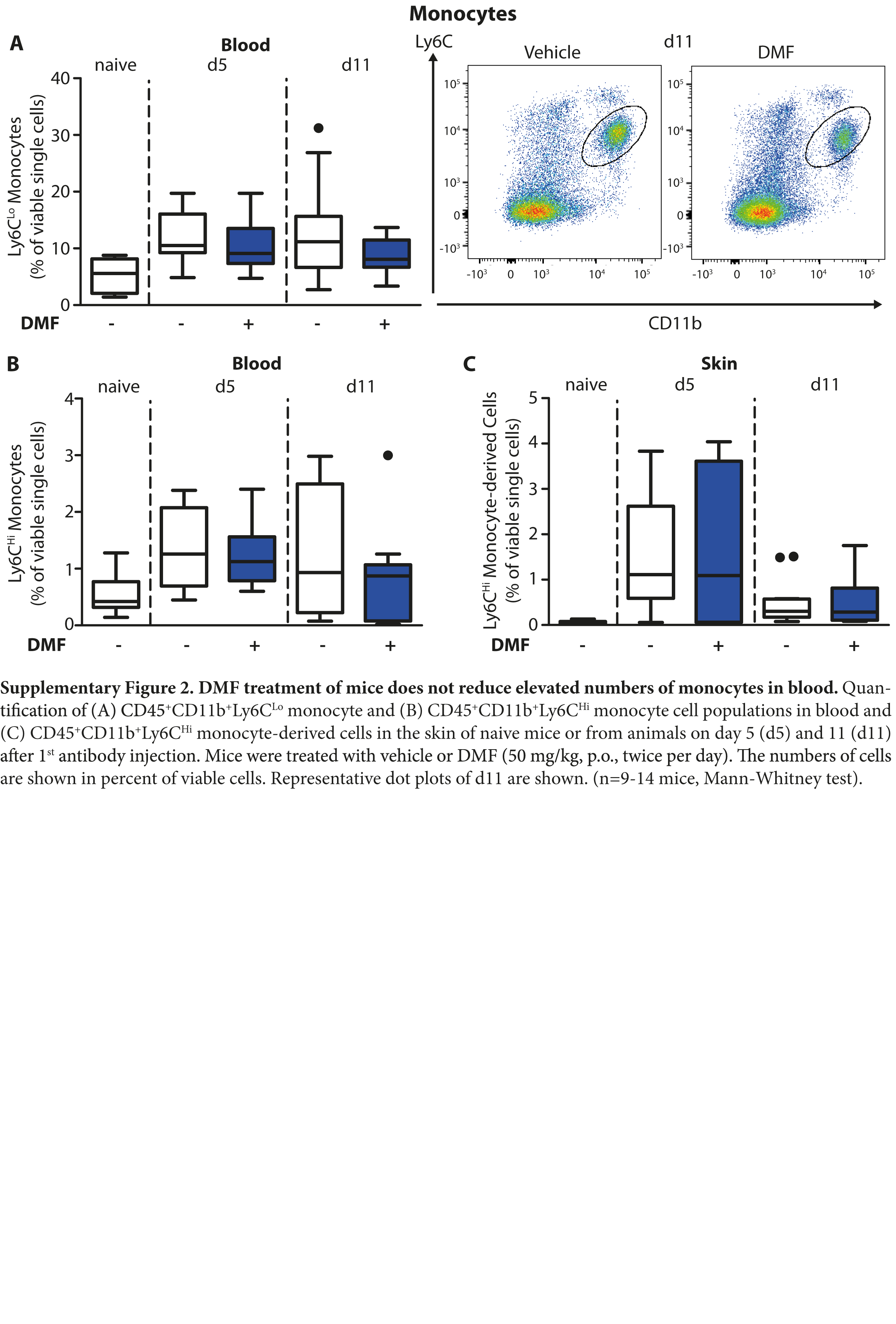

Supplement: Supplementary file 2 [file image_2.tif]

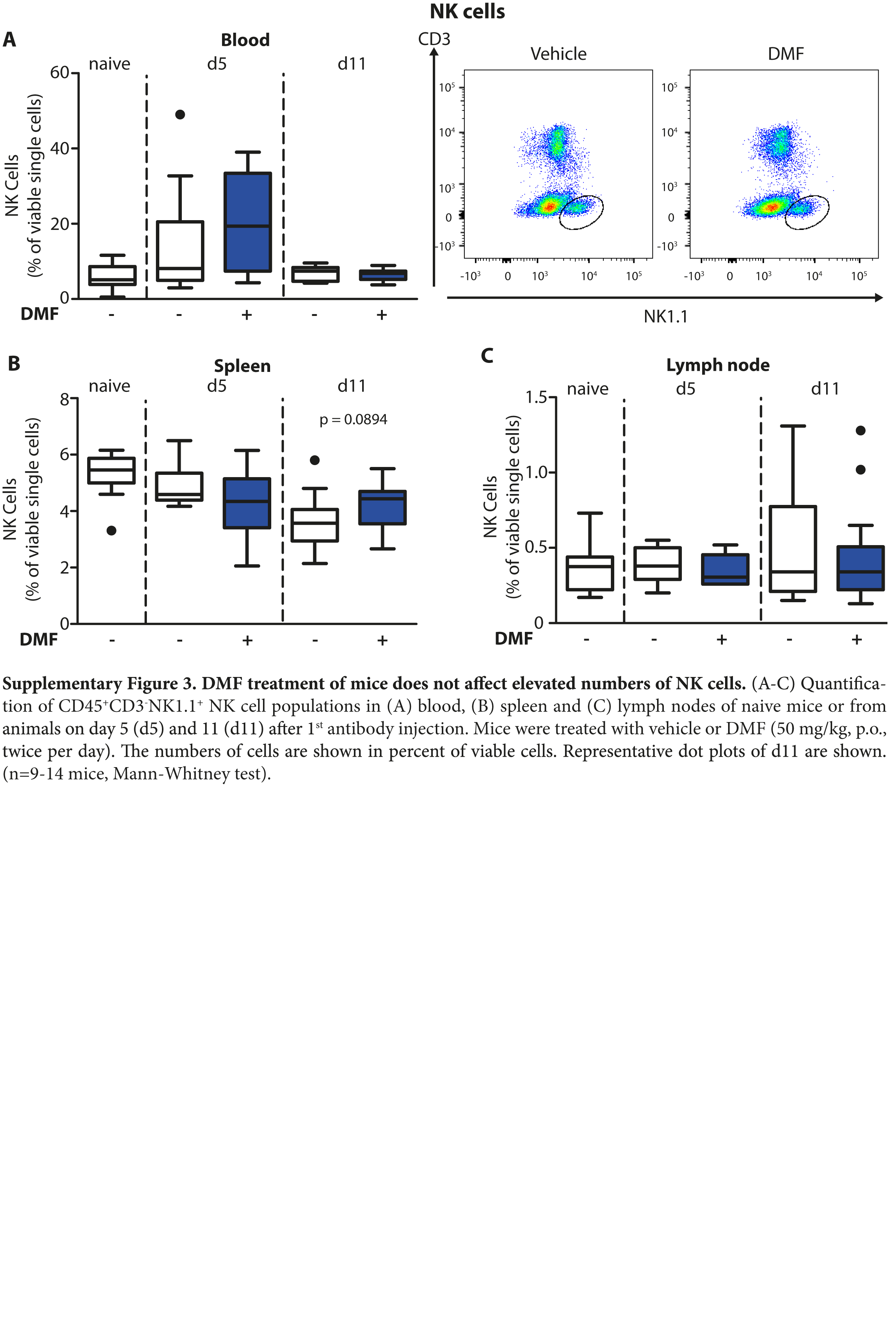

Supplement: Supplementary file 3 [file image_3.tif]

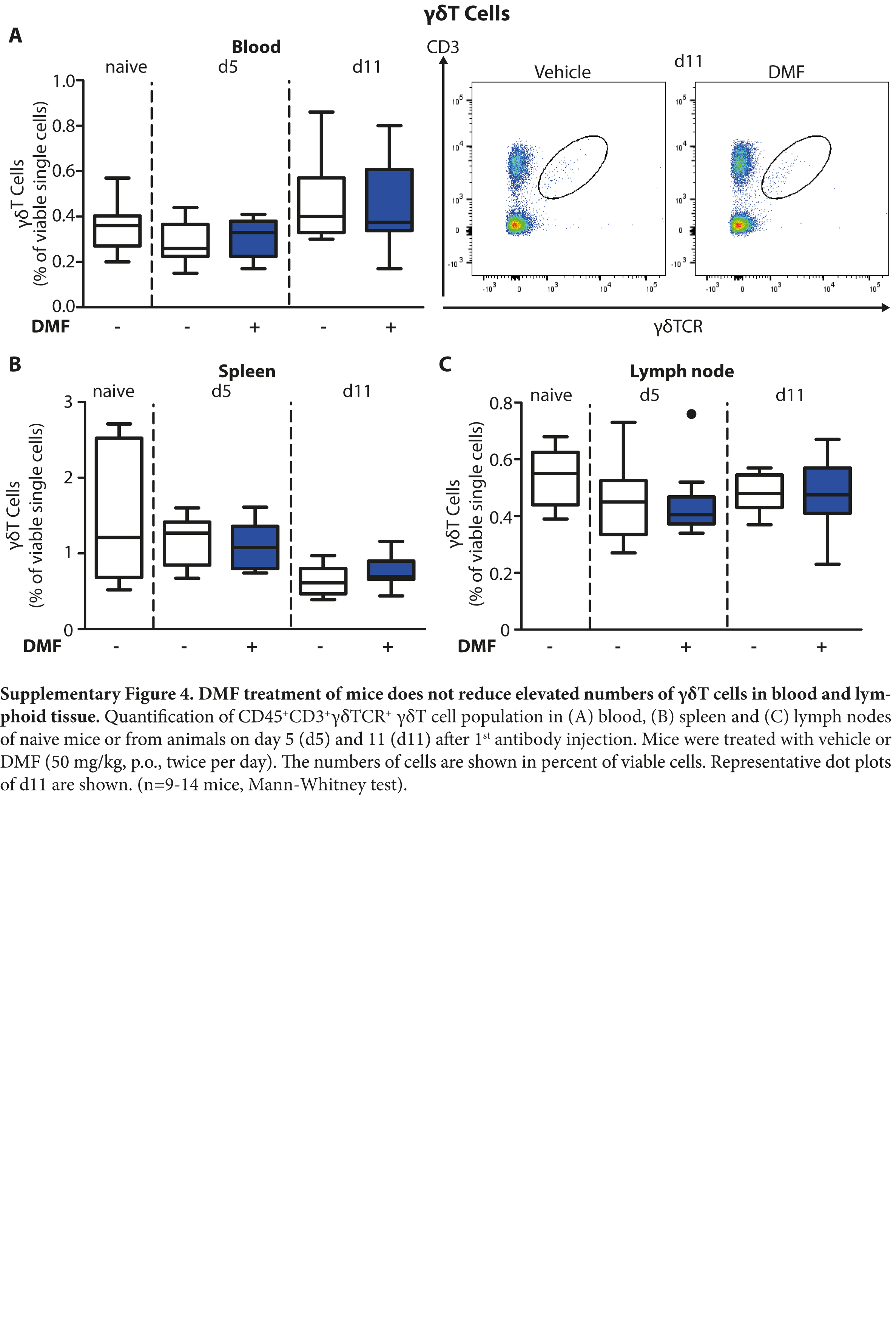

Supplement: Supplementary file 4 [file image_4.tif]

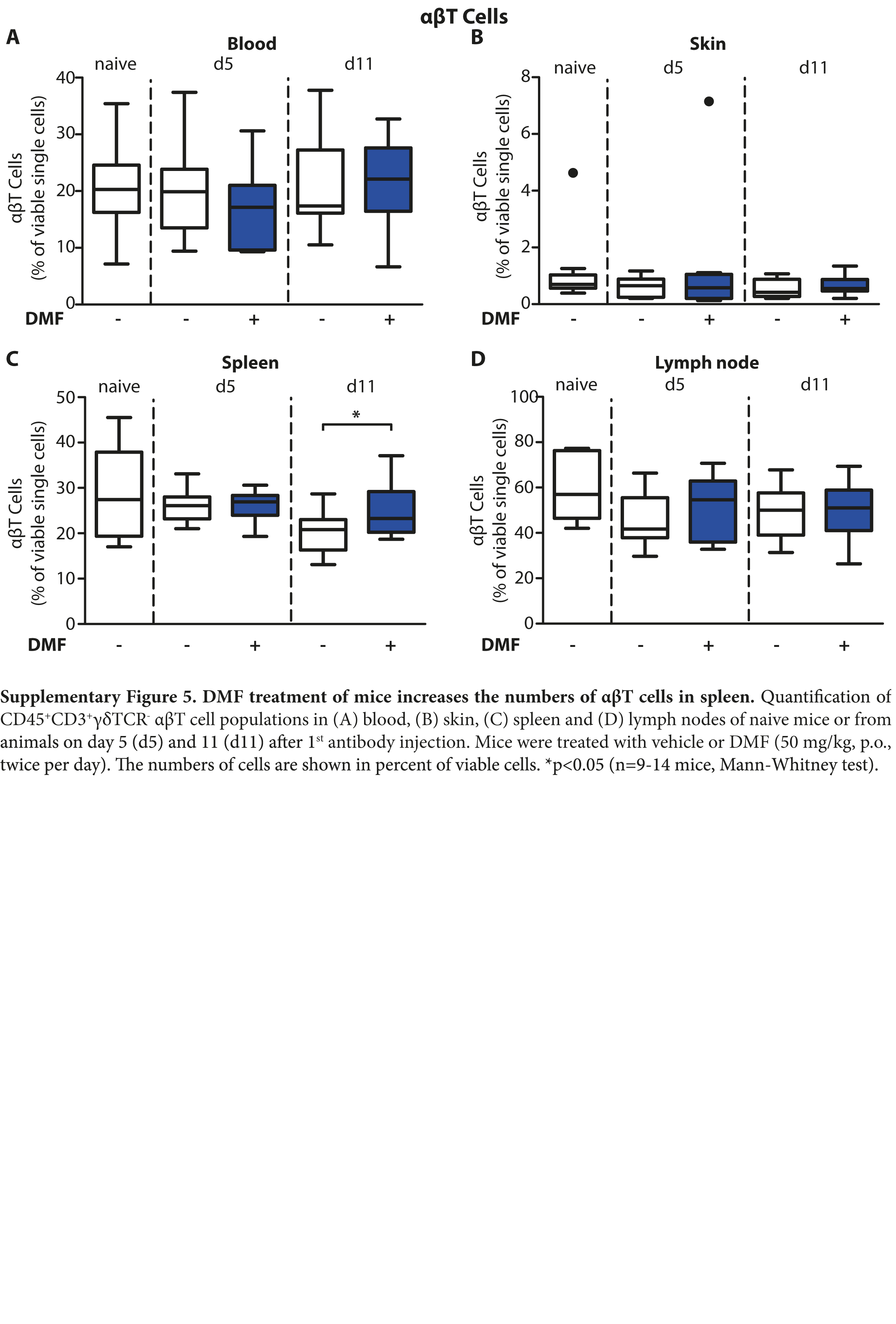

Supplement: Supplementary file 5 [file image_5.tif]

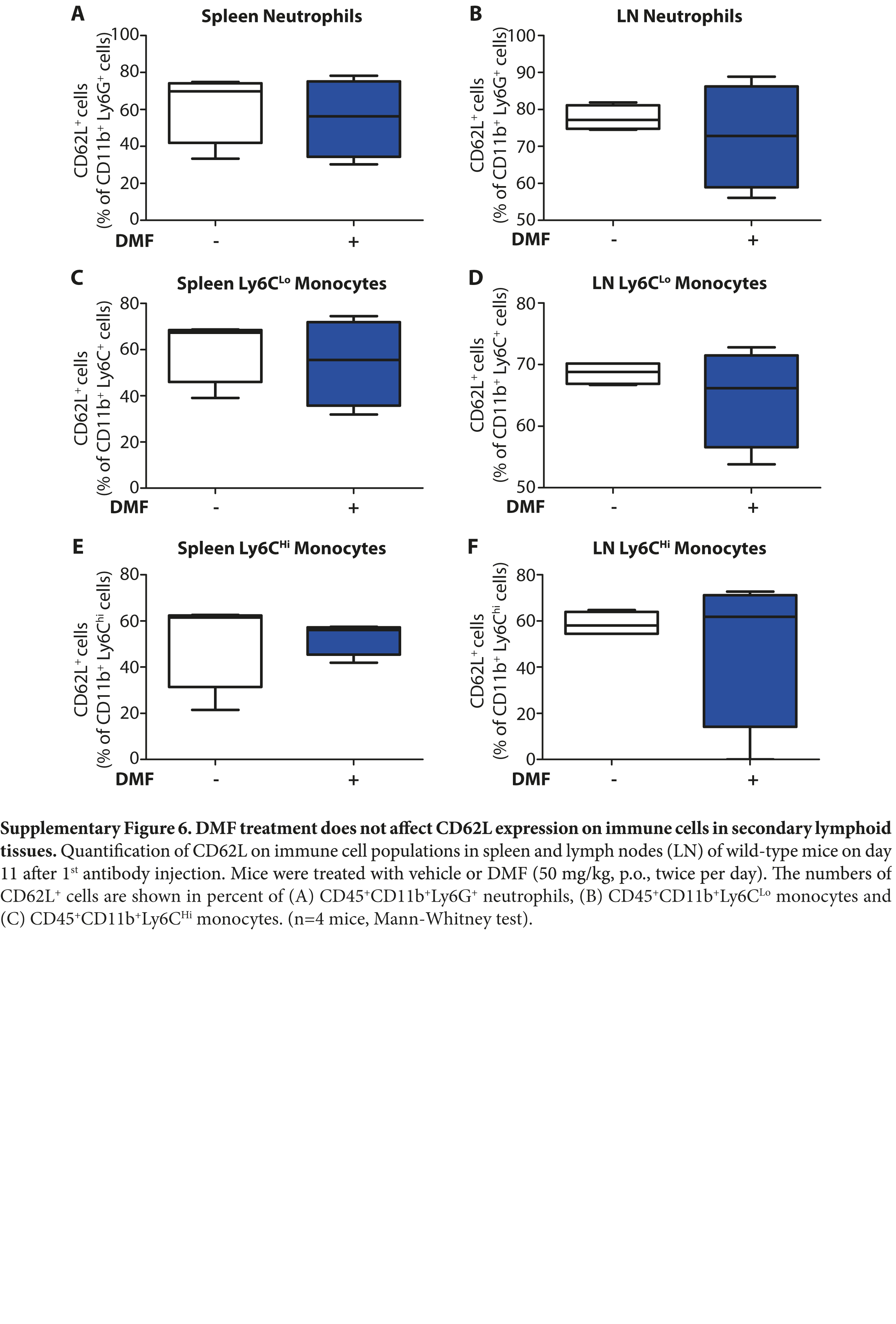

Supplement: Supplementary file 6 [file image_6.tif]

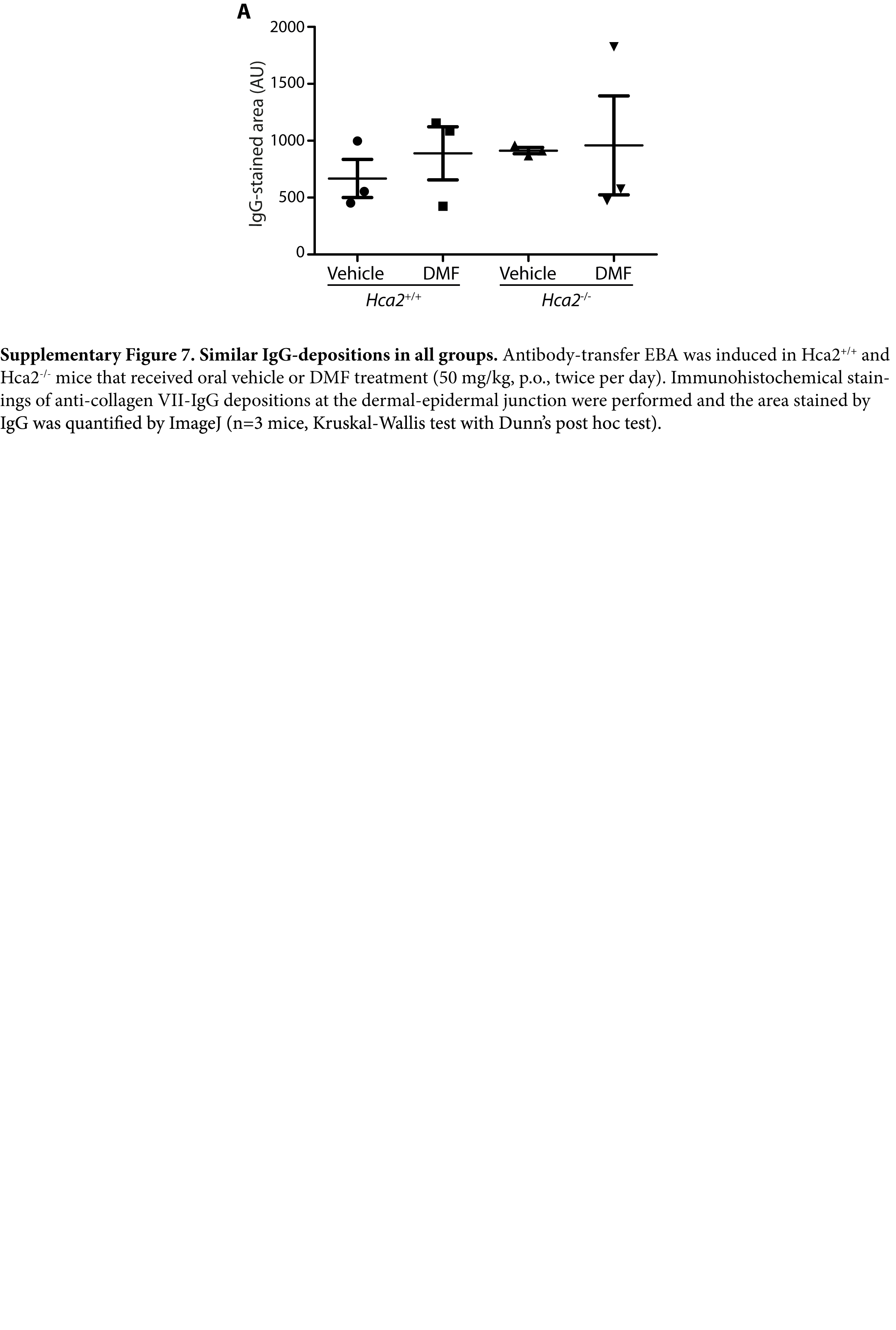

Supplement: Supplementary file 7 [file image_7.tif]

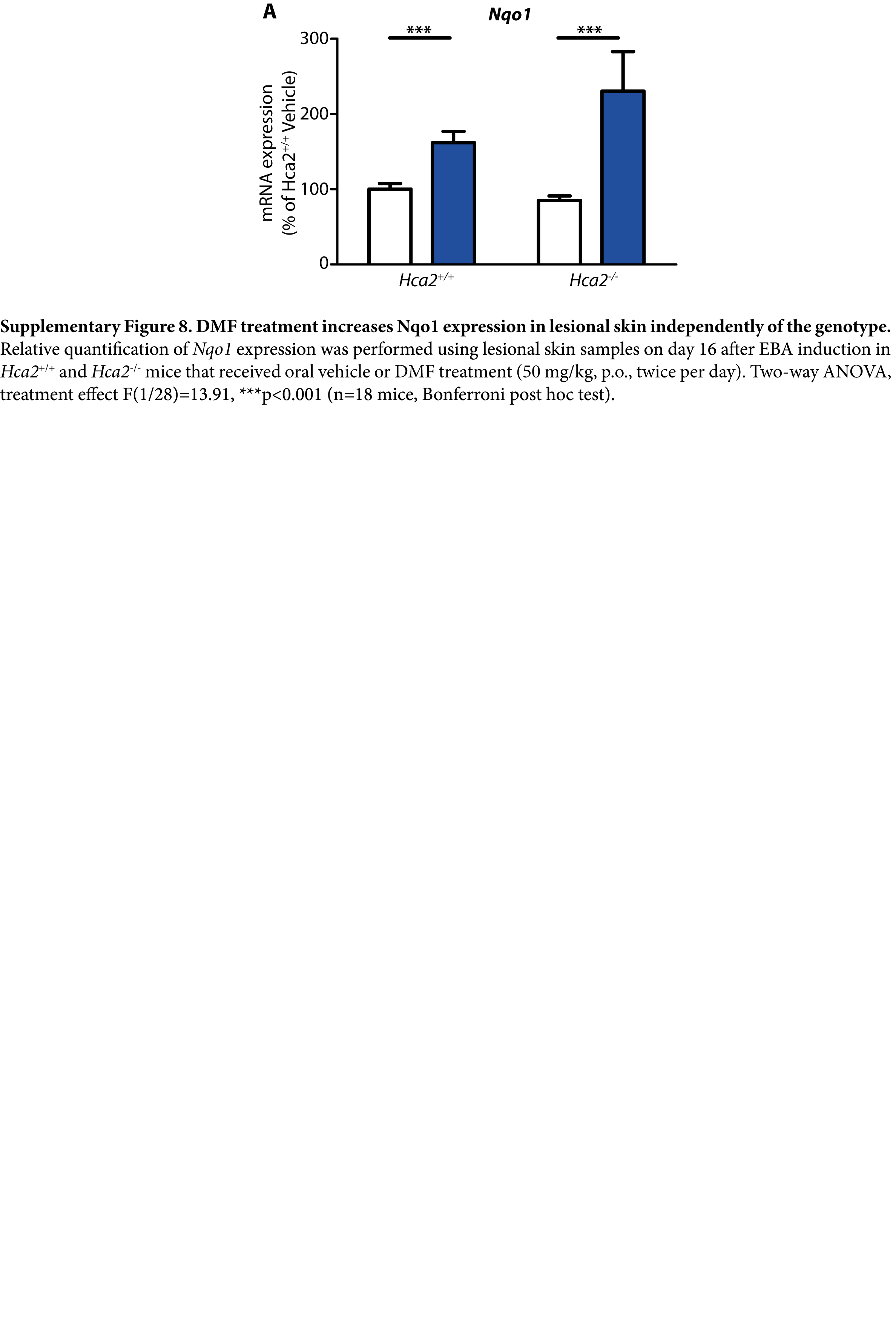

Supplement: Supplementary file 8 [file image_8.tif]

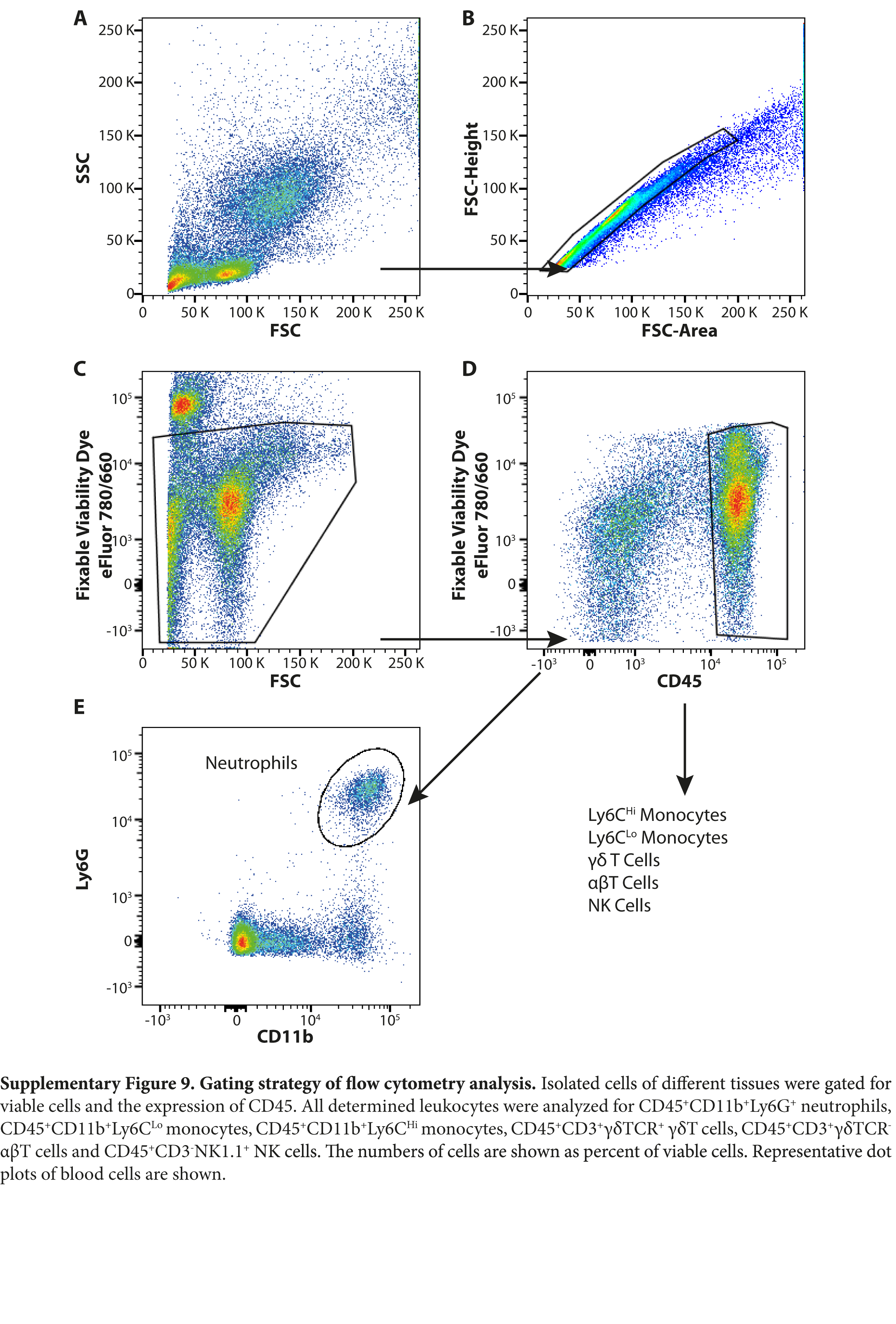

Supplement: Supplementary file 9 [file image_9.tif]
